# Comparison of capillary microsampling and venous blood for multi-pathogen serosurveillance

**DOI:** 10.1038/s41598-026-61174-6

**Published:** 2026-07-06

**Authors:** Rose Ombati, Makobu Kimani, Donald Akech, Bernadette Kutima, Antipa K. Sigilai, Boniface Karia, Victor Osoti, Angela Karani, Abdhalah Ziraba, Godfrey Bigogo, Patrick Munywoki, J. Anthony G. Scott, Ambrose Agweyu, E. Wangeci Kagucia, James Nyagwange

**Affiliations:** 1 KEMRI-Wellcome Trust Research Programme, Kilifi, Kenya; 2 African Population and Health Research Center, Nairobi, Kenya; 3 KEMRI Centre for Global Health Research, Kisumu, Kenya; 4 Division for Global Health Protection, Center for Global Health, US Centers of Disease Control and Prevention, Nairobi, Kenya; 5 Department of Infectious Diseases Epidemiology, London School of Hygiene and Tropical Medicine, London, UK; 6 Kenya Medical Research Institute (KEMRI)-Wellcome Trust Research Programme, P.O. Box 230, Kilifi, 80108 Kenya

**Keywords:** Serosurveillance, DBS, Multiplex immunoassay, IgG antibodies, Vaccine-preventable diseases, Antibody stability, Diseases, Health care, Immunology, Medical research, Microbiology

## Abstract

Serosurveillance for vaccine-preventable diseases (VPDs) can inform public health strategies by identifying gaps in immunization programs. However, venous blood sampling, though reliable and sensitive for serosurveillance, presents logistical challenges in resource-limited settings. Capillary microsampling using dried blood spots (DBS) offers a simpler, less invasive alternative that reduces cold-chain and personnel requirements. This study evaluated the performance and stability of DBS collected on filter paper and Mitra microsamplers compared with venous plasma. IgG antibody levels against diphtheria, tetanus, pertussis, measles, mumps, rubella, and varicella were quantified using a validated fluorescent bead-based multiplex immunoassay. At baseline, strong agreement was observed between DBS and plasma, with ≥ 93% of observations within the 95% limits of agreement. Sensitivity was high (≥ 95.8%) for all pathogens except pertussis (72.2–77.8%). For DBS stored at − 20 °C with desiccants, agreement remained high at 90 days, with gradual declines observed beyond one year. At room temperature, IgG levels declined, with sensitivity ≥ 91.2% at 7 and 30 days but dropping below 90% by 90 days for several analytes. Beyond one year, IgG recovery was minimal, with sensitivity < 50% for most pathogens. These findings support DBS utility for VPD serosurveillance, with stability up to 90 days at − 20 °C and 30 days at room temperature.

**Supplementary Information:**

The online version contains supplementary material available at https://doi.org/10.1038/s41598-026-61174-6.

## Introduction

Serosurveillance can provide insights into population immunity and population-level pathogen exposure to inform disease prevention measures including immunization program optimization^1^. Efficient serosurveillance requires rapid, cost-effective, sensitive, and reliable methodologies. However, in prospective population-based serological surveys, the most resource-intensive phases involve sample collection, shipment, processing, and storage of serological samples^2,3^. Serum or plasma from venous blood are regarded as the gold-standard specimens for serosurveillance. However, venous blood collection is invasive. In addition, it requires trained personnel and specialized equipment for immediate processing, cold-chain storage, and shipment. These requirements pose logistical challenges, particularly in remote and low-resource settings, where disease burden and susceptibility are less known^4^.

Capillary blood microsampling using dried blood spots (DBS) offers a practical alternative sampling approach for prospective population-based serosurveillance. DBS simplifies sample collection, reduces reliance on highly trained personnel, and can reduce the demands for cold-chain shipment and storage^2,3,5,6^. However, conventional DBS filter papers are associated with drawbacks, such as undefined blood volume and hematocrit variations, which can affect analytical accuracy. Advances in microsampling technologies have led to the development of devices such as Mitra volumetric absorptive microsamplers (VAMS)^7,8^, which are designed to collect a defined blood volume, potentially reducing the hematocrit bias associated with the processing of samples directly spotted on DBS filter paper^7–10^. Although few studies have directly compared different capillary microsampling approaches, Mitra devices have shown high sensitivity and specificity (≥ 95%) for anti-SARS-CoV-2 IgG detection, demonstrating the highest clinical sensitivity among different microsampling devices^11^.

Although DBS samples are commonly used as index specimens for detecting antibodies and other biomarkers in low-resource settings^12^, studies report considerable variability in both analytical methods and reported results^3,13–16^. Few studies have compared the performance of DBS with serum or plasma samples. In addition, the effects of storage temperature and duration on antibody integrity in DBS have not been systematically evaluated using well-matched reference specimens. Furthermore, there are no standardized elution methods or assay protocols for quantifying antibody concentrations in DBS. These limitations hinder the development of serosurveillance protocols for DBS collection and testing that ensure consistent and reliable antibody recovery.

In this study, we compared the performance of DBS collected on filter papers and Mitra microsamplers with venous blood plasma for the serosurveillance of seven vaccine-preventable diseases (VPDs) using a well-validated multiplex platform^17–19^. We aimed to optimize and validate the DBS elution protocol, compare IgG antibody concentrations and serostatus between DBS and paired plasma samples (as reference standard), and evaluate antibody recovery in DBS samples stored at freezing (− 20 °C) and room temperature (RT) over different time points.

## Results

### Study population demographics

We recruited 263 participants, with a nearly equal distribution across the three study sites: Kilifi HDSS (*n* = 86; 32.7%), Nairobi Urban HDSS (*n* = 87; 33.1%), and Manyatta HDSS (*n* = 90; 34.2%). Overall, 132 participants (50.2%) were male. The median age was 29 years (interquartile range: 15–45.5). The detailed age distribution of participants is presented in Table 1. The number of paired plasma and DBS samples collected across study sites, storage conditions (room temperature [RT] or frozen), and elution time points for immunoassay analysis is summarized in Supplementary Fig. S2. To assess potential site-related differences in antibody distributions, we performed sensitivity analysis comparing plasma IgG concentrations across the three HDSS sites using age-adjusted linear regression models (Supplementary Table S1). Significant site-level differences were observed for diphtheria, tetanus, and varicella, whereas no statistically significant differences were detected for pertussis, measles, mumps, or rubella. Plasma IgG-age scatterplots stratified by site (Supplementary Fig. S3) showed broadly comparable age-related IgG patterns across the three study populations, without evidence of major systematic shifts in antibody distributions.

**Table 1:** Number of study participants sampled by HDSS site, sex, and age category.

HDSS site	Kilifi *n* = 86	Nairobi Urban *n* = 87	Manyatta *n* = 90	Total *N* = 263
Sex				
Female	46 (53.5%)	31 (35.6%)	55 (61.1%)	132 (50.2%)
Male	40 (46.5%)	56 (64.4%)	35 (38.9%)	131 (49.8%)
Age categories				
< 5 years	0 (0%)	3 (3.4%)	4 (4.4%)	7 (2.7%)
5–14 years	10 (11.6%)	19 (21.8%)	26 (28.9%)	55 (20.9%)
15–24 years	12 (14%)	16 (18.4%)	21 (23.3%)	49 (18.6%)
25–34 years	11 (12.8%)	14 (16.1%)	18 (20.0%)	43 (16.3%)
35–44 years	12 (14%)	11 (12.6%)	12 (13.3%)	35 (13.3%)
45–54 years	18 (20.9%)	14 (16.1%)	5 (5.6%)	37 (14.1%)
55–64 years	15 (17.4%)	8 (9.2%)	3 (3.3%)	26 (9.9%)
≥ 65 years	8 (9.3%)	2 (2.3%)	1 (1.1%)	11 (4.2%)

Values are presented as n (%).

### Concordance of IgG antibody levels between DBS at baseline and matched plasma samples

**Fig. 1:**
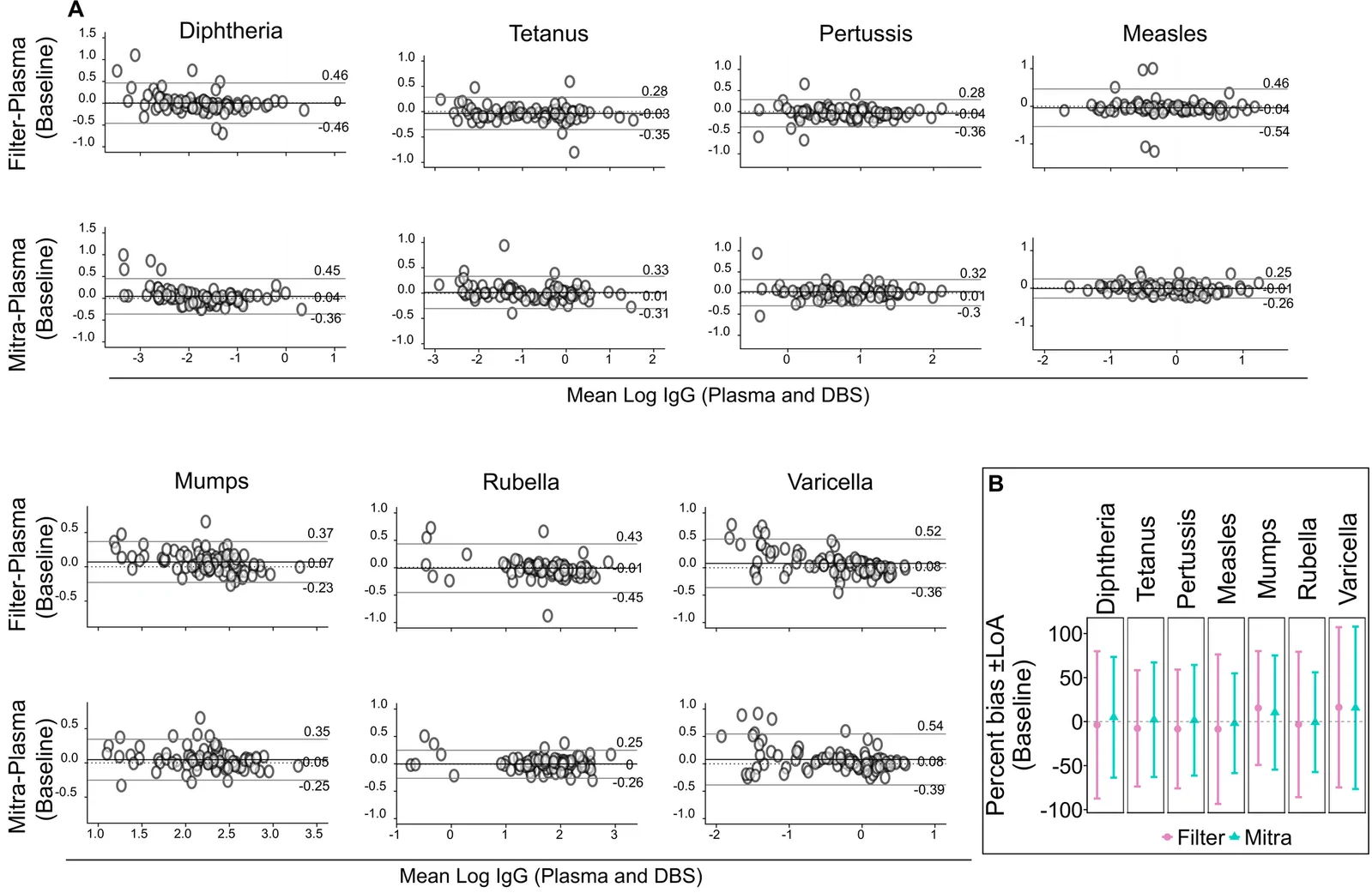
Comparison of IgG antibody levels in DBS samples at baseline (day 0) with matched plasma. (**A**) Bland-Altman plots showing agreement between log-transformed IgG responses measured in plasma and DBS filter paper (top) or DBS Mitra (bottom) at baseline (*n* = 85) across seven analytes: diphtheria, tetanus, pertussis, measles, mumps, rubella, and varicella. The solid black line represents the mean difference (average bias), and the grey dashed lines denote the 95% limits of agreement (mean ± 1.96 SD). The dotted horizontal line at zero indicates ideal agreement. Numerical values for the mean bias and upper and lower limits of agreement are displayed on the right margin of each plot. (**B**) Faceted Bland-Altman summary plots illustrating the mean percent bias and 95% limits of agreement for DBS matrices (filter paper and Mitra) compared with plasma. Points represent mean percent bias, with vertical error bars showing the upper and lower limits of agreement. The horizontal dashed line at zero indicates perfect agreement. Filter paper data are shown as pink circles, and Mitra data as blue triangles.

To evaluate baseline performance, IgG concentrations from DBS samples eluted within 24 h of collection were compared with matched plasma samples as the reference standard. Bland-Altman analyses demonstrated strong agreement across all seven analytes, with ≥ 93% of paired observations falling within the 95% limits of agreement (LoA). Mean biases were minimal. Slight positive biases were observed for mumps (filter paper: 0.07; Mitra: 0.05) and varicella (0.08 for both matrices), reflecting marginally higher concentrations in DBS (Fig. 1A). Percent bias was similarly low, ranging from − 8.6% (measles) to 16.2% (varicella) for filter paper and − 1.8% (measles) to 15.6% (varicella) for Mitra (Fig. 1B). Correlation analyses confirmed strong linearity between DBS and plasma IgG concentrations across all analytes (*R* ≥ 0.90). Concordance correlation coefficients (CCC ≥ 0.90) also indicated high concordance, with slightly lower values for diphtheria (CCC = 0.87) and tetanus (CCC = 0.88) on filter paper (Supplementary Fig. S4).

Diagnostic performance based on binary serostatus classification showed high sensitivity (≥ 95.8%) for all pathogens except pertussis (filter: 72.2%; Mitra: 77.8%). Specificity was also high (≥ 92.3%) for most analytes but was lower for measles (72.7%), mumps (filter: 89.0%), rubella (filter: 70.0%, Mitra: 88.9%), and varicella (filter: 89.3%) (Supplementary Table S2).

### Reduced IgG antibody levels upon freeze-storage of DBS samples

**Fig. 2:**
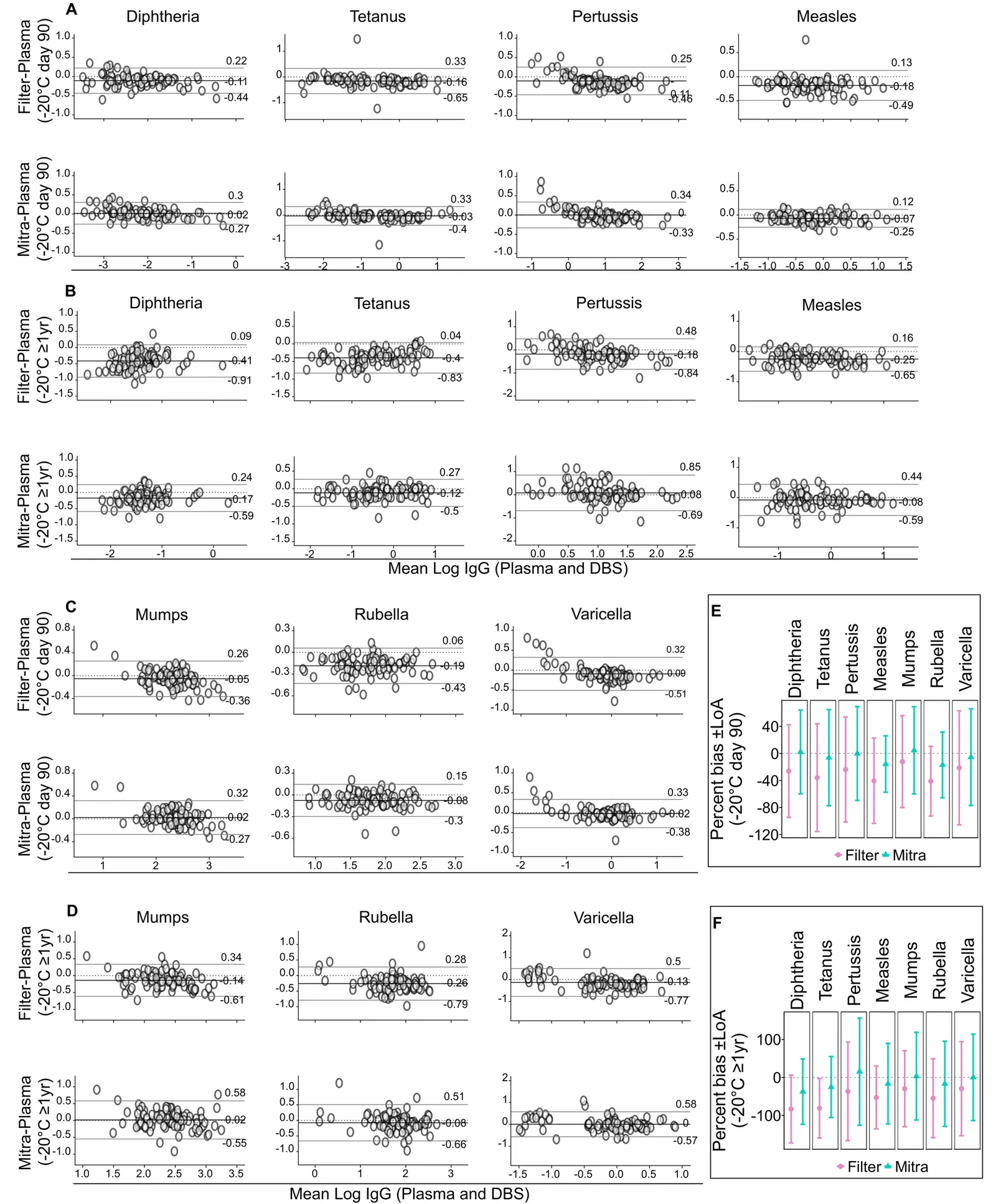
Comparison of IgG antibody levels in DBS samples stored at − 20 °C relative to matched plasma samples. Bland-Altman plots showing agreement between log-transformed IgG responses quantified in plasma and DBS filter paper (top panels) or Mitra (bottom panels) after storage at − 20 °C for 90 days (*n* = 87) and > 1 year (*n* = 88). (**A**,** B**) Diphtheria, tetanus, pertussis, and measles at 90 days and > 1 year, respectively. (**C**,** D**) Mumps, rubella, and varicella at 90 days and > 1 year, respectively. The solid black line represents the mean difference, and the grey dashed lines indicate the 95% LoA (mean ± 1.96 SD). The dotted line at zero denotes ideal agreement. Numerical values for the mean bias and upper and lower LoA are shown along the right margin of each plot. (**E**,** F**) Faceted Bland-Altman summary plots illustrating mean percent bias and 95% LoA for DBS matrices (filter paper and Mitra) compared with plasma for the 90-day and > 1-year time points, respectively. Points represent mean percent bias, with vertical error bars indicating the upper and lower LoA. Filter paper data are shown as pink circles, and Mitra data as blue triangles. The horizontal dashed line at zero indicates perfect agreement.

To assess antibody stability during frozen storage, we compared IgG concentrations from DBS stored at − 20 °C with matched plasma at − 80 °C at two time points: day 90 and > 1 year (459–496 days). This is because baseline DBS and plasma responses were comparable, and plasma under ultra-cold storage does not vary considerably from baseline responses over time. At day 90, agreement remained high, with < 7/87 (< 8.0%) of observations outside the LoA. Mean biases were minimal, ranging from − 0.05 (mumps) to − 0.19 (rubella) for filter paper, and from 0 (pertussis) to − 0.08 (rubella) for Mitra (Fig. 2A, C). Percent bias remained modest across most analytes, but measles and rubella showed larger negative bias in filter paper (− 40.4% to − 41.1%) (Fig. 2E).

After > 1 year, bias increased for filter paper ranging from − 0.13 (varicella) to − 0.41 (diphtheria), while Mitra retained lower bias (0 for varicella to − 0.17 for diphtheria) (Fig. 2B, D). Percent bias widened, particularly for filter paper, ranging from 3.3% (mumps) to − 83.2% (diphtheria) (Fig. 2F).

Correlations remained high at day 90 (*R* ≥ 0.93), with high CCC values for Mitra (≥ 0.94 except diphtheria 0.88; mumps 0.83) and more variable for filter paper (0.62–0.89). After > 1 year, correlations remained high, but CCC declined, especially for filter paper (pertussis 0.45; rubella 0.39; varicella 0.24), reflecting reduced antibody recovery over time (Supplementary Fig. S5).

At day 90, sensitivity was ≥ 90.9% except for pertussis (filter: 62.5%; Mitra: 87.5%). Specificity was ≥ 93.9% for diphtheria, pertussis and varicella, but reduced for tetanus (filter: 87.5%; Mitra: 62.5%) and mumps (filter: 75.0%), measles (Mitra: 83.3%), and rubella (Mitra: 66.7%) (Supplementary Table S3). At > 1 year, sensitivity remained ≥ 90.1% for most pathogens but declined for measles (81.6%) and diphtheria (77.0%) in filter paper and markedly for pertussis (filter: 33.3%; Mitra: 71.9%). Specificity remained (≥ 91.2%) for filter paper but declined for Mitra, particularly for measles (63.6%) and mumps (0.0%). Specificity could not be calculated for tetanus, as all plasma and corresponding DBS samples were seropositive (Supplementary Table S4). Overall, DBS samples were stable for up to 90 days at − 20 °C, with notable declines thereafter, especially in filter paper.

### Storage of DBS samples at room temperature reduces IgG antibody levels

**Fig. 3:**
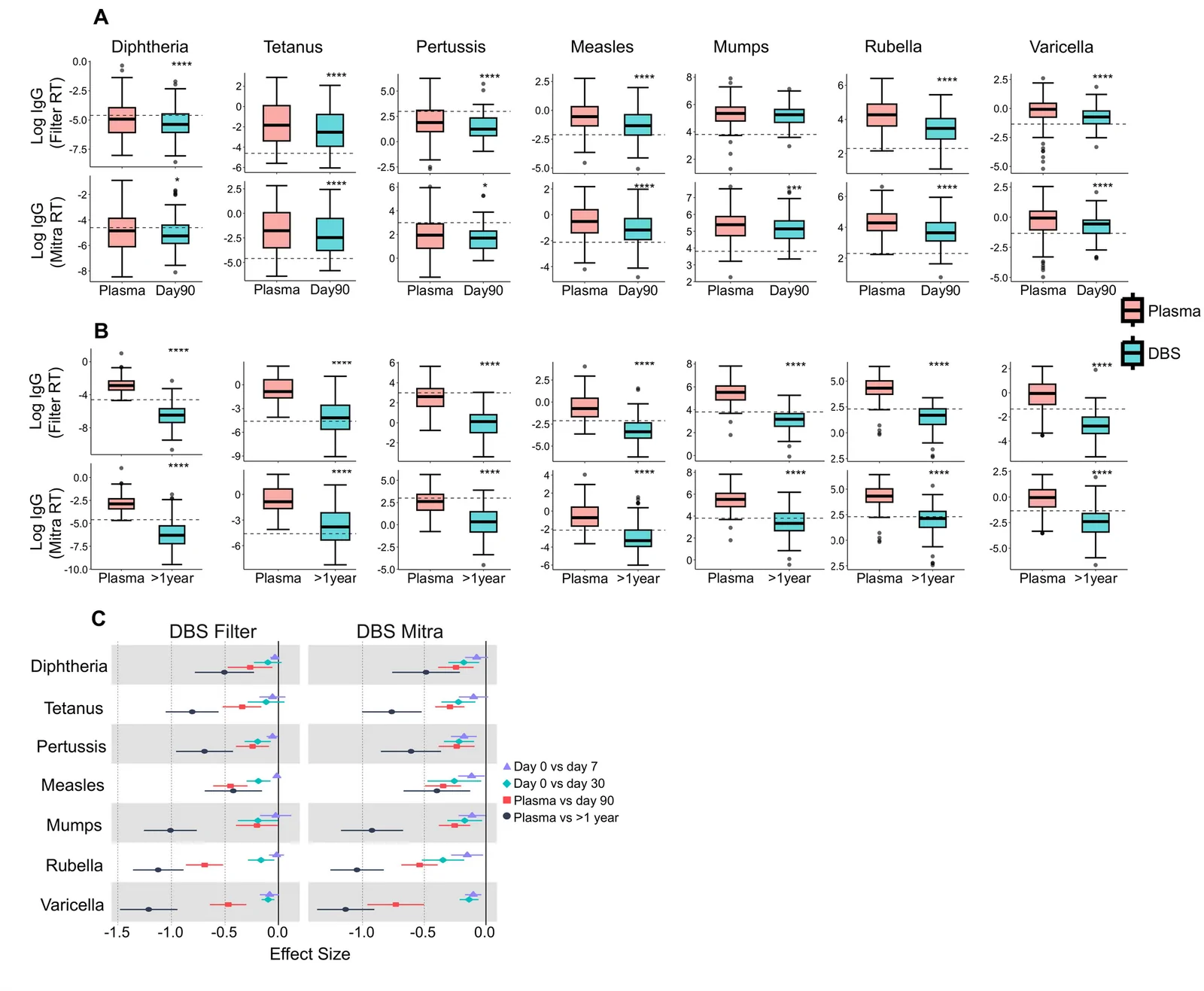
Analysis of IgG antibody levels in DBS samples stored at room temperature (RT) across various time points. (**A**,** B**) Log-transformed antibody concentrations from DBS filter (upper panel) and Mitra (lower panel) eluates at day 90 (*n* = 87) (**A**) and > 1 year (*n* = 90) (**B**) compared with paired plasma levels. The line in each box represents the median, and the box shows the interquartile range (IQR). Whiskers indicate the range excluding outliers (> 1.5× IQR). Black dotted lines mark pathogen-specific seropositivity cutoffs. Asterisks indicate statistical significance (*p < 0.05, **p < 0.01, ***p < 0.001, ****p < 0.0001). (**C**) Forest plot showing effect sizes with 95% confidence intervals derived from pairwise contrasts estimated from the mixed-effects model for antibody levels in DBS filter and Mitra samples. Day 7 and 30 levels are compared with baseline (day 0), while day 90 and > 1 year are compared with matched plasma.

Antibody stability under ambient conditions was evaluated for DBS stored at room temperature for 7, 30, and 90 days, and > 1 year. IgG levels at days 7 and 30 were compared with baseline (day 0), whereas day-90 and > 1-year eluates were compared with plasma stored at − 80 °C for the same durations. IgG concentrations remained stable at day 7. A slight decline was observed by day 30, most notably for measles and rubella; however, these changes were not statistically significant across matrices (*p* ≥ 0.05) (Supplementary Fig. S6). By day 90, IgG concentrations had decreased significantly for most pathogens, except mumps on filter paper (*p* ≥ 0.05) (Fig. 3A). After > 1 year, IgG concentrations were markedly reduced for all pathogens and both matrices (*p* < 0.0001) (Fig. 3B). Effect sizes summarizing these changes are shown in Fig. 3C and Supplementary Table S5. Overall, antibody levels declined progressively across storage time points, with the highest decline observed after > 1 year.

At day 7, sensitivity was ≥ 95.2% for all pathogens except pertussis (filter: 71.4%; Mitra: 66.7%), with high specificity (mostly 100%), with lower values for tetanus (83.3%) and measles (80.0%) on filter paper (Supplementary Table S6). By day 30, sensitivity remained ≥ 91.2% for most pathogens but was lower for pertussis (filter: 75.0%; Mitra: 60.0%). Specificity ranged from 85.7% to 100% across most analytes (Supplementary Table S7). By day 90, sensitivity declined below 90.0% for diphtheria, pertussis, measles (Mitra and filter paper), rubella (filter paper), and varicella (Mitra), whereas specificity remained high (≥ 93.3%) except for tetanus and mumps (≤ 75.0%) (Supplementary Table S8). After > 1 year, sensitivity dropped below 50% for most pathogens except tetanus (filter: 57.5%; Mitra: 69.0%) (Supplementary Table S9).

## Discussion

This study evaluated DBS microsampling as an alternative to venous blood for the serosurveillance of VPDs (diphtheria, tetanus, pertussis, measles, mumps, rubella, and varicella) using a multiplex immunoassay platform. We assessed two DBS matrices, filter paper and Mitra devices, across multiple storage conditions to determine their viability for multi-pathogen serosurveillance. Elution protocols were optimized for both matrices to ensure consistent antibody recovery across seven analytes. While previous studies have primarily tailored protocols to single-analyte assays^3,18,20–23^, multiplex platforms require conditions that support uniform antibody recovery across targets. A 6 mm filter paper punch yielded approximately 4.5 µL of plasma with an optimal elution volume of 900 µL, comparable to plasma diluted at 1:200. While earlier estimates suggest 5.8–6.4 µL plasma per punch^24^, variability in blood spotting, viscosity, and hematocrit may account for these differences^25^. Matrix effects inherent to filter paper^26^ further underscore the need for assay-specific optimization of elution protocols. In contrast, Mitra devices collected a defined 30µL whole-blood volume per tip, corresponding to approximately 15 µL of plasma, with optimal elution at 3000 µL. By minimizing hematocrit-related bias, VAMS provides more consistent analyte recovery^6,8,26^, supporting its potential utility for multi-pathogen serosurveillance.

Sample stability is a key consideration for field implementation. We observed strong concordance between DBS and plasma when samples were processed within 24 h, with high sensitivity (≥ 95.0%) for all pathogens except pertussis. Frozen storage at − 20 °C preserved antibody stability for up to 90 days, consistent with previous studies reporting stable measles, mumps and rubella antibodies at both 4 °C and − 20 °C conditions for periods ranging from 120 days to six months^27,28^. However, storage beyond one year was associated with reduced antibody recovery, particularly in filter paper samples for diphtheria, tetanus, pertussis, mumps, and measles. These findings suggest that prolonged frozen storage may affect antibody recovery for some analytes and may require matrix-specific considerations.

Room temperature storage resulted in a progressive decline in antibody recovery. IgG concentrations at 7 and 30 days remained comparable to baseline, maintaining high sensitivity and minimal serostatus misclassification. By 90 days, significant declines were observed for most analytes, with corresponding reductions in diagnostic performance. After > 1 year, sensitivity fell below 50% for all pathogens except tetanus, indicating that extended ambient storage is unsuitable at 90 days and beyond. These findings align with previous studies reporting that prolonged ambient storage compromises measles, mumps, and rubella antibody integrity beyond 120 days^28^. Nevertheless, short-term room temperature storage (≤ 30 days) may remain feasible for time-sensitive applications such as outbreak investigations or rapid serosurveys. The observed decline during prolonged ambient storage may reflect heat-induced protein degradation or fixation to the DBS matrix. Elevated temperatures can induce protein denaturation and aggregation, thereby impairing antigen-binding capacity^29,30^. We also observed incomplete erythrocyte lysis in prolonged storage, with residual erythrocyte components visibly fixed to the filter paper after the overnight elution incubation. This observation suggests heat-mediated fixation of biomolecules to the DBS matrix, limiting antibody recovery. These findings highlight the importance of freezing DBS samples for long-term antibody preservation. However, further studies should explore alternative recovery methods to minimize the need for cold-chain storage, as ambient-temperature transport and storage remain more cost-effective and logistically feasible for large-scale serosurveillance studies.

Comparing the two microsampling methods, Mitra devices demonstrated reduced variability, stronger concordance with plasma, and higher sensitivity, particularly for storage for > 1 year at − 20 °C. The defined volumetric sampling and reduced hematocrit bias likely account for these performance advantages^31^. While filter paper showed declining antibody recovery after 90 days of frozen storage, particularly for diphtheria, tetanus, measles, and rubella, Mitra samples remained largely stable. Nonetheless, practical considerations such as cost and environmental footprint associated with single-use plastic should be weighed when selecting optimal sampling approaches.

Several aspects of the study design strengthen the validity and field applicability of these findings. The matched comparison of DBS and venous plasma samples enabled a direct evaluation of analytical performance across sampling matrices, ensuring that observed differences reflected the sampling method rather than population variability. Inclusion of three HDSS sites broadened geographic representation and enhanced the generalizability of findings across diverse field settings. Standardized training ensured consistent sample collection procedures across sites while centralized laboratory testing minimized analytical variability. The use of a multiplex immunoassay platform enabled simultaneous assessment of multiple pathogens, demonstrating the scalability of DBS microsampling for multi-pathogen serosurveillance.

This study has some limitations. The study relied on convenience sampling, which may limit the representativeness and introduce selection bias. The study population was predominantly adults, resulting in a high proportion of seropositive individuals with antibody concentrations well above the seropositivity cut-offs for pathogens such as tetanus and mumps. This skewed antibody distribution may inflate sensitivity estimates while reducing the precision of specificity estimates. Pertussis antibody concentrations were generally low, consistent with rapid waning of vaccine-induced immunity within three years post-vaccination^32,33^. In such low-prevalence settings, specificity is particularly important, and it remained relatively high despite reduced sensitivity. DBS samples were assigned to different storage durations by site, resulting in unequal distribution across time points. In addition, baseline DBS measurements were unavailable for two sites, where comparisons were made using matched plasma samples. Although baseline DBS and plasma concentrations were comparable, and plasma is generally stable under ultra-cold storage, the confounding between study site, age distribution and storage duration may have influenced estimates at 90 days and > 1 year. However, sensitivity analyses adjusting for age showed no statistically significant site-level differences in plasma IgG concentrations for most analytes, except diphtheria and tetanus, suggesting that underlying population heterogeneity alone is unlikely to account for the observed long-term DBS stability patterns. Future studies should include more balanced age distributions to better assess serostatus across pathogens with age-dependent immunity. Incorporating models of antibody decay may further improve seroprevalence estimates by accounting for reduced antibody recovery over time.

Overall, our findings support the feasibility of DBS microsampling as a practical alternative to venous blood collection for multi-pathogen serosurveillance using multiplex immunoassays, particularly in field or resource-limited settings, provided storage conditions are optimized. Early processing within 24 h, short-term room temperature storage for up to 30 days, and long-term frozen storage for > 1 year, particularly for Mitra devices, are viable approaches for the utility of DBS for serosurveillance. However, filter paper samples appear more susceptible to reduced antibody recovery during prolonged frozen storage (> 1 year), which should be considered in study design. Although we did not conduct a formal cost analysis, our experience suggests substantial cost differences between the DBS microsampling methods assessed; such practical aspects should be balanced against technical considerations when designing future serosurveillance.

## Methods

### Study design and participants

This study was nested within the Kenya Multi-site Integrated Serosurveillance (KEMIS) study during its third round of cross-sectional surveys, conducted from July to August 2023 at three Health and Demographic Surveillance System (HDSS) sites: Kilifi (KHDSS), Nairobi Urban (NUHDSS) and Manyatta (MHDSS)^34^. Detailed characteristics of these HDSS sites have been previously described^35–37^. Serosurveys at KHDSS and NUHDSS recruited a random, age-stratified sample, whereas MHDSS used random household sampling to enroll eligible residents from selected households. Approximately 850 participants were recruited at each site, as detailed in previous survey rounds^38,39^. For this study, a convenient sample of approximately 10% of survey participants at each site was targeted for enrollment.

Study participants aged ≥ 18 years provided written informed consent, while for those < 18 years, written consent was obtained from parents/guardians, with written assent for children aged 13–17 years. All experiments were performed in accordance with relevant guidelines and regulations. Ethical approval was obtained from the Kenya Medical Research Institute Scientific and Ethics Review Unit (KEMRI/SERU/CGMR-C/203/4085).

### Sample collection

All field staff across the HDSS sites received standardized training on sample collection procedures. Venous blood was collected using BD Vacutainer^®^ sodium heparin tubes, while capillary blood samples were obtained by finger pricking with a single-use lancet device and collected onto Whatman 903 filter paper cards (Whatman, Sanford, CA, USA) and Mitra^®^ devices (Neoteryx, Torrance, CA) (Supplementary Fig. S1). Matched sets of triplicate samples, consisting of venous blood, capillary DBS on filter paper, and Mitra, were collected from each participant. The DBS samples were air-dried at ambient temperature for 1 h and then packed in seal-top plastic bags with desiccants then transported to the site laboratories for interim storage prior to subsequent shipment to the KEMRI-Wellcome Trust Research Programme laboratory in Kilifi, Kenya for testing.

Matched DBS filter paper and Mitra samples were assayed at different time points to evaluate the impact of freezing and room temperature (RT, 20–25 °C) storage on antibody concentrations against the seven VPDs. Baseline (Day 0) samples from the KHDSS site were eluted within 24 h of collection; this was only feasible at this site due to its proximity to the testing laboratory. A second set of DBS samples was stored at RT and eluted at 7 and 30 days (KHDSS), 90 days (NUHDSS), and beyond the 1-year time point (459–496 days; MHDSS). A third set was stored at − 20 °C and eluted at 90 days (NUHDSS) and beyond the 1-year time point (MHDSS). For KHDSS, IgG concentrations at subsequent time points were compared with corresponding baseline DBS responses. At NUHDSS and MHDSS sites, where immediate elution was not feasible due to site-level limitations, DBS IgG concentrations were compared with the corresponding plasma responses, which were considered the reference standard.

### Sample processing

Plasma was isolated from venous whole blood by centrifugation at 1300 g for 10 min at 18–25 °C, then stored at − 80 °C until analysis. For DBS filter paper samples, a 6 mm diameter punch, equivalent to ~ 12 µL of whole blood, was excised from the centre (saturated portions of the dried spot) using a BSD600 Ascent puncher (BSD Robotics) into a microcentrifuge tube. In the case of filter papers with variable spot sizes, punches were taken from spots with a minimum diameter of 6 mm to ensure a fully saturated sample. For DBS Mitra samples, the microsampler tips, containing ~ 30 µL of whole blood, were detached directly into separate microcentrifuge tubes.

A standard protocol was used to elute DBS samples. Each DBS filter paper punch and Mitra tip was separately incubated overnight at 2–8 °C on a shaker in assay buffer comprising phosphate-buffered saline with 0.1% Tween 20 and 3% bovine serum albumin^15,40^. The eluate was transferred into a microcentrifuge tube and centrifuged at 15,000 rpm for 15 min. The supernatant was then collected and briefly stored at − 20 °C until the assay run.

The optimal elution volume for DBS samples was determined through in-house optimization by comparing IgG levels in DBS eluates and matched plasma. Eluting a 6 mm filter paper punch in 900 µL of assay buffer and a Mitra tip in 3000 µL showed the highest concordance with plasma samples diluted 1:200. This elution protocol was subsequently used for all DBS samples.

### Multiplex immunoassay

IgG antibody concentrations against diphtheria, tetanus, pertussis, measles, mumps, rubella, and varicella-zoster were quantified using a fluorescent bead-based multiplex immunoassay on the Luminex platform, as previously described^17–19,41^. Matched sets of plasma, DBS filter paper, and Mitra samples were assayed in duplicate. Seropositivity was defined using the standard cut-offs for the multiplex immunoassay: diphtheria ≥ 0.01 IU/mL, tetanus ≥ 0.01 IU/mL, pertussis ≥ 20 IU/mL, measles ≥ 0.12 IU/mL, mumps ≥ 45 RU/mL, rubella ≥ 10 IU/mL, and varicella ≥ 0.26 IU/mL.

### Statistical analysis

To evaluate site‑related differences in antibody distributions, log‑transformed plasma IgG concentrations were analyzed using linear regression with HDSS site as the main predictor and age included as a covariate; fold‑changes were derived from model coefficients. Age–IgG patterns were visualized using site‑stratified scatterplots. The agreement between IgG antibody levels measured in DBS samples (filter paper and Mitra devices) and matched plasma (reference standard) was evaluated using Bland-Altman analysis. Serostatus was classified using antigen-specific IgG cut-off values, and sensitivity and specificity were calculated by comparing DBS-based serostatus with the corresponding reference (plasma or baseline DBS). Supplementary analyses examined the association between quantitative IgG levels in DBS and plasma using both the Pearson correlation coefficient (R) and the concordance correlation coefficient (CCC). To assess the stability of IgG concentrations in DBS samples stored at room temperature, log-transformed analyte-specific IgG levels were plotted across time points to visualize trends in concentration decay. A linear mixed-effects regression model was then fitted to estimate the change in IgG levels over time, with storage duration modelled as a categorical fixed effect and participant ID specified as a random effect to account for variation due to individual differences. Pairwise contrasts between storage durations were estimated from the fitted mixed-effects models. Statistical significance was defined as *p* < 0.05. All analyses were performed using R version 4.3.1 and R Studio 2024.09.0^42^.

## Supplementary Information

Below is the link to the electronic supplementary material.

Supplementary Material 1

Supplementary Material 2

## Data Availability

The datasets and R scripts used for the analysis in this study are available from the corresponding author upon reasonable request.
