# Supplementary material for: Comparison of capillary microsampling and venous blood for multi-pathogen serosurveillance
Source: Sci Rep. 2026 Jul 6;16:31046. doi: 10.1038/s41598-026-61174-6 (PMC13638452; doi:10.1038/s41598-026-61174-6)
Supplement: Supplementary file 1 [file 41598_2026_61174_MOESM2_ESM.pdf]

Comparison of capillary microsampling and venous blood for multi-pathogen serosurveillance

Supplementary Figures

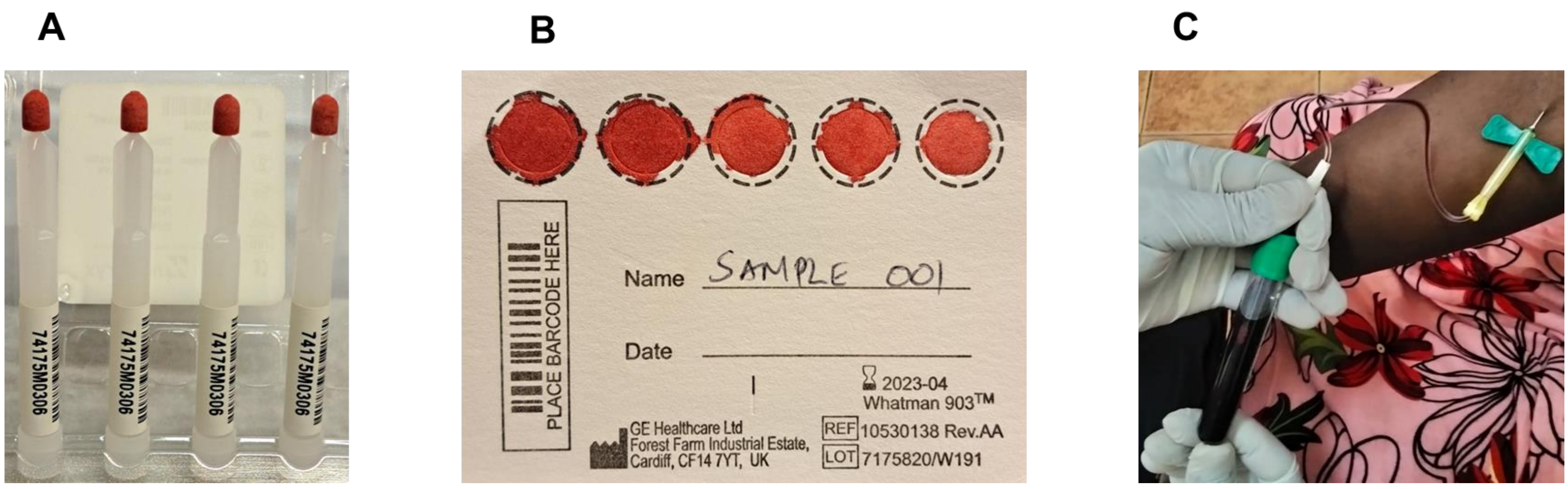

Figure S1: Blood sample collection tools used in the study: (**A**) Mitra® microsampling devices with blood samples collected via finger prick. (**B**) Whatman 903 filter paper card spotted with dried blood for DBS collection. (**C**) Venous blood collection performed using BD Vacutainer® sodium heparin tubes. Capillary blood was collected through a finger prick, while venous blood was drawn via standard venipuncture.

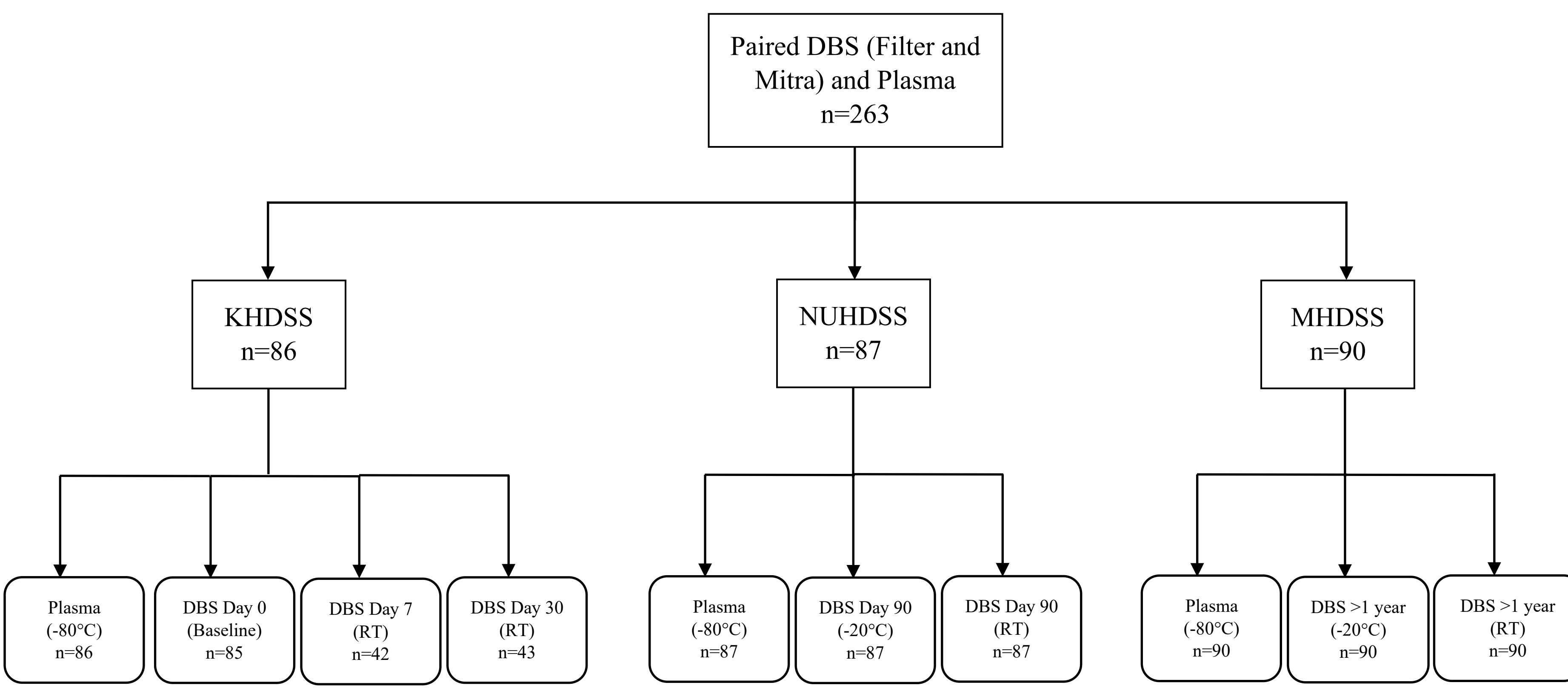

Figure S2: Collection of paired plasma and DBS samples using filter paper and Mitra devices. The DBS sample sizes collected from Kilifi (KHDSS), Nairobi Urban (NUHDSS) and Manyatta (MHDSS) sites are shown, along with storage conditions (room temperature (RT) or frozen) and corresponding elution time points for immunoassay analysis.

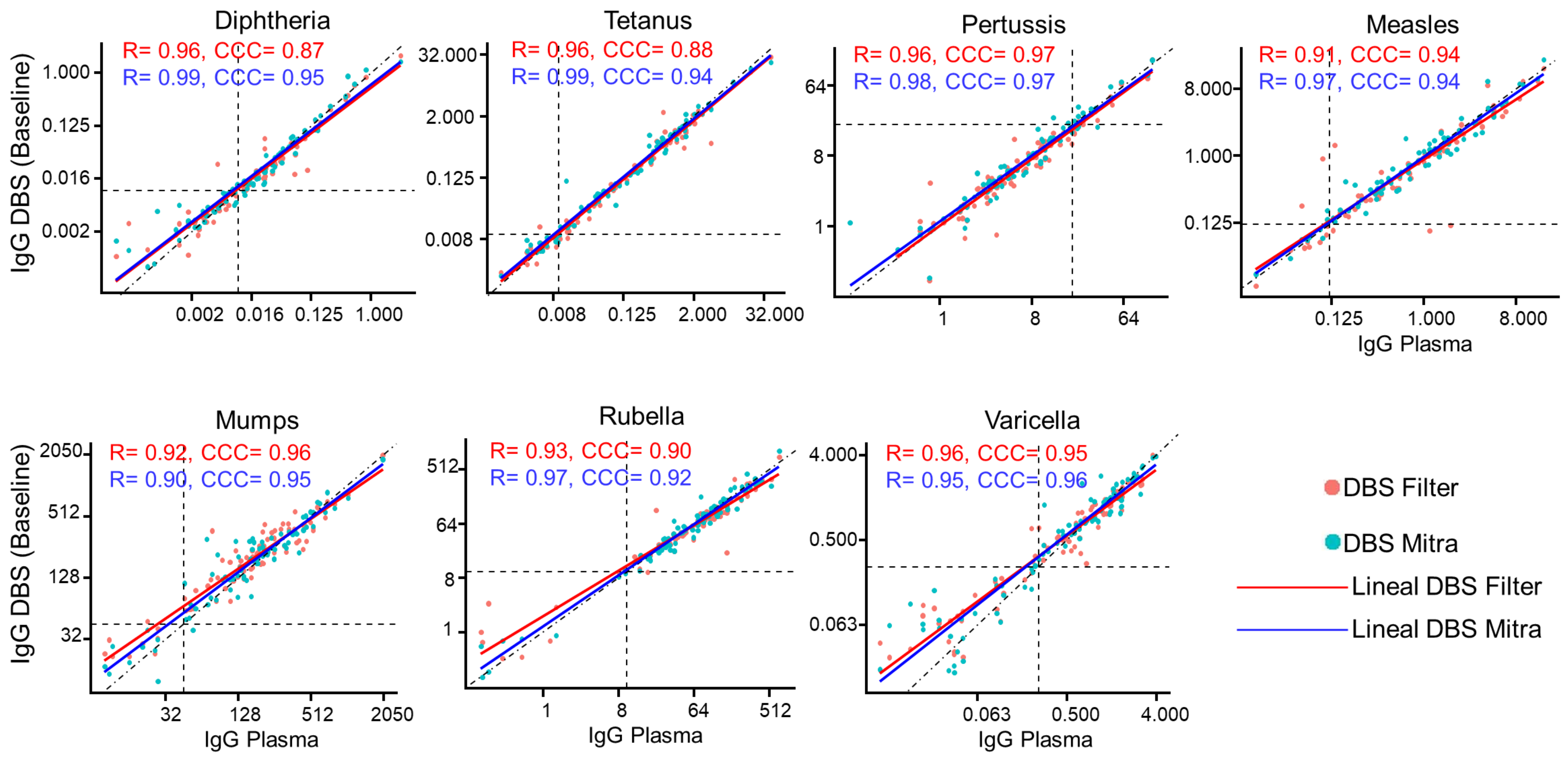

Figure S3: Comparison of IgG antibody levels in DBS samples at baseline (day 0) with matched plasma. Correlation analysis of IgG concentrations in baseline DBS filter paper (red) and Mitra (blue) samples relative to plasma. The dotted diagonal line represents the reference lines (x=y), while the horizontal and vertical black dashed lines indicate the pathogen-specific seropositivity cut-off thresholds. Corresponding Pearson correlation coefficients (R) and concordance correlation coefficients (CCC) are shown.

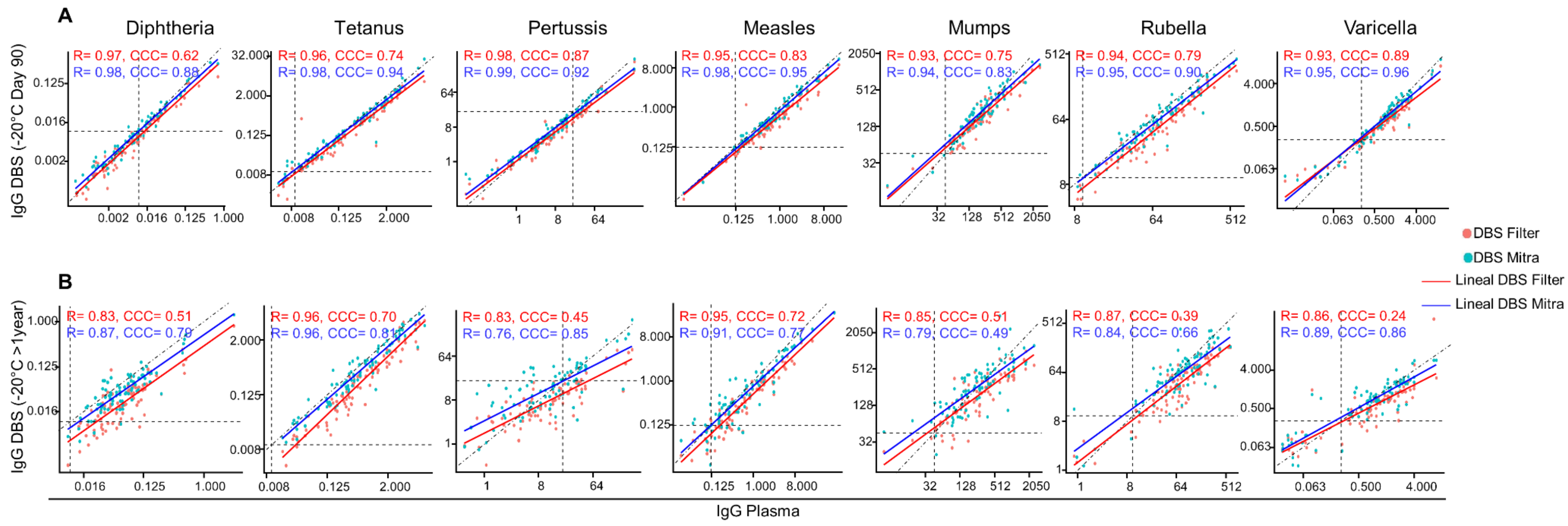

Figure S4: Comparison of IgG antibody levels in DBS samples stored at -20°C relative to matched plasma samples. Correlation plots showing IgG concentrations quantified in DBS filter paper (red points and regression lines) and DBS Mitra (blue) compared with plasma for seven analytes: diphtheria, tetanus, pertussis, measles, mumps, rubella, and varicella. The diagonal dotted line represents the x = y reference line. Horizontal and vertical black dashed lines indicate analyte-specific seropositivity cut-off thresholds. Panel **(A)** shows results from samples stored for 90 days, while panel **(B)** presents results from samples stored for >1 year. Pearson correlation coefficients (R) and concordance correlation coefficients (CCC) for each analyte and sample type are shown.

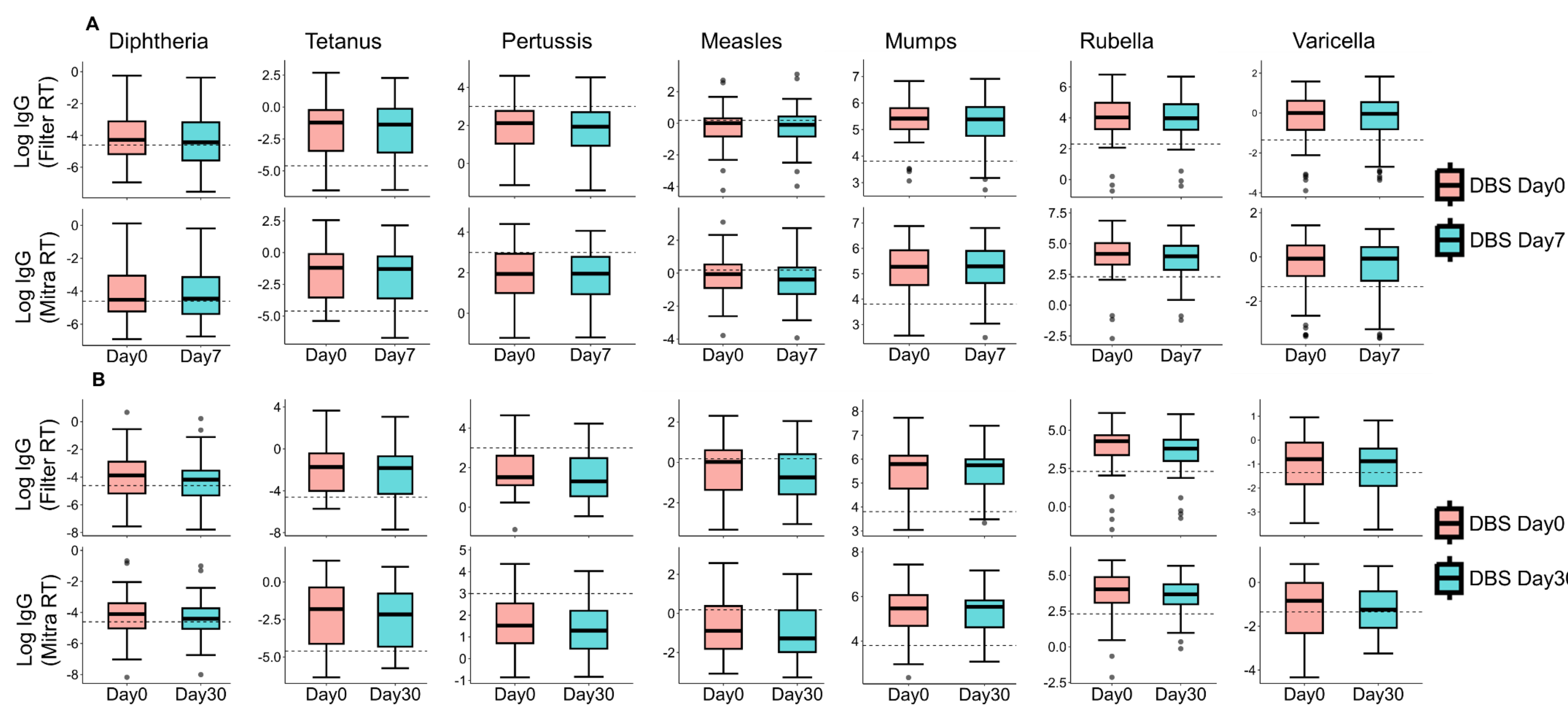

Figure S5: Analysis of IgG antibody levels in DBS samples stored at room temperature (RT) across various time points. Log-transformed antibody concentrations from DBS filter (upper panel) and Mitra (lower panel) eluates at RT day 7 (**A**) and at day 30 (**B**) compared with paired plasma levels. The line in each box represents the median, and the box shows the interquartile range (IQR). Whiskers indicate the range excluding outliers (>1.5× IQR). Black dotted lines mark pathogen-specific seropositivity cutoffs.

Supplementary Tables

Table S1: Sensitivity and Specificity of DBS filter and Mitra at baseline (Day 0) relative to plasma IgG responses

Analyte	DBS Filter Sensitivity	DBS Filter Specificity	DBS Mitra Sensitivity	DBS Mitra Specificity
Diphtheria	100% (93.2−100)	93.9% (79.8−99.3)	96.2% (86.8−99.5)	93.9% (79.8−99.3)
Tetanus	95.8% (88.6−99.1)	92.3% (64.0−99.8)	98.6% (92.5−100)	100% (75.3−100)
Pertussis	72.2% (46.5−90.3)	100% (94.6−100)	77.8% (52.4−93.6)	97.0% (89.6−99.6)
Measles	96.0% (88.6−99.2)	72.7% (39.0−94.0)	100% (95.1−100)	72.7% (39.0−94.0)
Mumps	100% (95.3−100)	89.0% (51.8−99.7)	98.7% (92.9−100)	100% (66.4−100)
Rubella	100% (95.2−100)	70.0% (34.8−93.3)	100% (95.3−100)	88.9% (51.8−99.7)
Varicella	100% (93.7−100)	89.3% (71.8−97.7)	100% (93.7−100)	96.4% (81.7−99.9)

Table S2: Sensitivity and Specificity of DBS filter and Mitra at stored at -20°C for 90 days relative to plasma IgG responses

Analyte	DBS Filter Sensitivity	DBS Filter Specificity	DBS Mitra Sensitivity	DBS Mitra Specificity
Diphtheria	90.9% (75.7−98.1)	100% (93.4−100)	93.9% (79.8−99.3)	94.4% (84.6−98.8)
Tetanus	100% (95.4−100)	87.5% (47.4−99.7)	100% (95.4−100)	62.5% (24.5−91.5)
Pertussis	62.5% (40.6−81.2)	100% (94.6−100)	87.5% (67.6−97.3)	100% (94.3−100)
Measles	93.8% (86.2−98.0)	100% (54.1−100)	96.3% (89.6−99.2)	83.3% (35.9−99.6)
Mumps	100% (95.7−100)	75% (19.4−99.4)	100% (95.7−100)	100% (39.8−100)
Rubella	98.8% (93.5−100)	100% (29.2−100)	100% (95.7−100)	66.7% (9.4−99.2)
Varicella	97.2% (90.3−99.7)	97.2% (90.3−99.7)	97.2% (90.3−99.7)	93.3% (68.1−99.8)

Table S3: Sensitivity and Specificity of DBS filter and Mitra at stored at -20°C for >1 year relative to plasma IgG responses.

Analyte	DBS Filter Sensitivity	DBS Filter Specificity	DBS Mitra Sensitivity	DBS Mitra Specificity
Diphtheria	77.0% (66.8−85.4)	100% (2.5−100.0)	95.4% (88.5−98.7)	100% (2.5−100.0)
Tetanus	97.6% (90.4−99.3)	N/A (0.0−100.0)	100.0% (95.9−100.0)	N/A (0.0−100.0)
Pertussis	33.3% (18.0−51.8)	100% (93.5−100.0)	71.9% (53.3−86.3)	85.5% (73.3−93.5)
Measles	81.6% (71.0−89.6)	91.2% (61.5−99.8)	92.1% (83.6−97.1)	63.6% (30.1−89.1)
Mumps	91.8% (83.8−96.6)	100% (29.2−100.0)	97.6% (91.7−99.7)	0.0% (0.0−70.8)
Rubella	96.4% (89.8−99.3)	100.0% (47.8−100.0)	100.0% (95.6−100.0)	80.0% (28.4−99.5)
Varicella	90.1% (80.7−95.9)	94.1% (71.3−99.9)	98.6% (92.3−100.0)	88.2% (63.6−98.5)

Table S4: Sensitivity and Specificity of DBS filter and Mitra at Day 7 room temperature storage relative to Day 0 time point IgG responses

Analyte	DBS Filter Sensitivity	DBS Filter Specificity	DBS Mitra Sensitivity	DBS Mitra Specificity
Diphtheria	95.2% (76.2−99.9)	100% (82.4−100)	95.5% (77.2−99.9)	100% (81.5−100)
Tetanus	97.1% (84.7−99.9)	83.3% (35.9−99.6)	100% (89.7−100)	100% (54.1−100)
Pertussis	71.4% (29.0−96.3)	100% (89.4−100)	66.7% (29.9−92.5)	100% (88.8−100)
Measles	100% (90.0−100)	80.0% (28.4−99.5)	97.3% (85.8−99.9)	100% (29.2−100)
Mumps	97.2% (85.5−99.9)	100% (39.8−100)	100% (90.3−100)	100% (39.8−100)
Rubella	97.2% (85.5−99.9)	100% (39.8−100)	97.2% (85.5−99.9)	100% (39.8−100)

Varicella	100% (89.1−100)	100% (63.1−100)	96.9% (83.8−99.9)	100% (63.1−100)

Table S5: Sensitivity and Specificity of DBS filter and Mitra at Day 30 room temperature storage relative to Day 0 time point IgG responses

Analyte	DBS Filter Sensitivity	DBS Filter Specificity	DBS Mitra Sensitivity	DBS Mitra Specificity
Diphtheria	96.4% (81.7−99.9)	100% (73.6−100)	92.0% (74.0−99.0)	100% (73.5−100)
Tetanus	91.2% (76.3−98.1)	100% (54.1−100)	100% (88.4−100)	71.4% (29.0−96.3)
Pertussis	75.0% (34.9−96.8)	96.9% (83.8−99.9)	60.0% (14.7−94.7)	100% (89.1−100)
Measles	96.9% (83.8−99.9)	62.5% (24.5−91.5)	97.3% (85.8−99.9)	100% (29.2−100)
Mumps	100% (90.3−100)	100% (39.8−100)	93.3% (77.9−99.2)	85.7% (42.1−99.6)
Rubella	100% (89.7−100)	100% (54.1−100)	100% (89.1−100)	100% (47.8−100)
Varicella	100% (85.8−100)	93.8% (69.8−99.8)	100% (83.2−100)	100% (80.5−100)

Table S6: Sensitivity and Specificity of DBS filter and Mitra at stored at Room Temperature (RT) for 90 days relative to plasma IgG responses

Analyte	DBS Filter Sensitivity	DBS Filter Specificity	DBS Mitra Sensitivity	DBS Mitra Specificity
Diphtheria	69.7% (51.3−84.4)	98.2% (90.1−100)	70.3% (53.02−84.1)	100% (92.9−100)
Tetanus	96.2% (89.3−99.2)	75.0% (34.9−96.8)	98.7% (93.2−100)	62.5% (24.5−91.5)
Pertussis	62.5% (40.6 − 81.2)	100% (94.6−100)	42.9% (21.8−66.0)	100% (94.6−100)
Measles	45.8% (25.6−67.2)	100% (94.3−100)	84.2% (74.4−91.3)	100% (47.8−100)
Mumps	100% (95.7−100)	75.0% (19.4−99.4)	96.4% (89.8−99.3)	50.0% (6.8−93.2)
Rubella	76.5% (65.8−85.3)	100% (54.1−100)	94.2% (87.0−98.1)	100% (2.5−100)
Varicella	97.2% (90.3−99.7)	97.2% (90.3−99.7)	88.9% (79.3−95.1)	93.3% (68.1−99.8)

Table S7: Sensitivity and Specificity of DBS filter and Mitra stored at RT for >1 year relative to plasma IgG responses

Analyte	DBS Filter Sensitivity	DBS Filter Specificity	DBS Mitra Sensitivity	DBS Mitra Specificity
Diphtheria	5.9% (1.9−13.2)	100% (2.5−100.0)	10.6% (5.0−19.1)	100.0% (2.5−100.0)
Tetanus	57.5% (46.4−68.0)	_% (0.0−100.0)	69.0% (58.1−78.5)	_% (0.0−100.0)
Pertussis	3.1% (0.1−16.2)	100.0% (93.5−100.0)	25.0% (11.5−43.4)	100.0% (93.5−100.0)
Measles	21.3% (12.7−32.3)	100.0% (73.5−100.0)	29.0% (19.1−40.5)	100.0% (71.5−100.0)
Mumps	19.1% (11.3−29.1)	100.0% (29.2−100.0)	34.2% (24.0−45.5)	66.7% (9.4−99.2)
Rubella	29.3% (19.7−40.4)	100.0% (47.8−100.0)	46.3% (35.3−57.7)	100.0% (47.8−100.0)
Varicella	10.0% (4.1−19.5)	100.0% (80.5−100.0)	24.3% (14.8−36.0)	100.0% (80.5−100.0)
