# Supplementary material for: Comparison of capillary microsampling and venous blood for multi-pathogen serosurveillance
Source: Sci Rep. 2026 Jul 6;16:31046. doi: 10.1038/s41598-026-61174-6 (PMC13638452; doi:10.1038/s41598-026-61174-6)
Supplement: Supplementary file 2 [file 41598_2026_61174_MOESM1_ESM.docx]

# **Comparison of capillary microsampling and venous blood for multi-pathogen serosurveillance**

**Supplementary Figures**

**Figure S1:** Blood sample collection tools used in the study: (**A**) Mitra® microsampling devices with blood samples collected via finger prick. (**B**) Whatman 903 filter paper card spotted with dried blood for DBS collection. (**C**) Venous blood collection performed using BD Vacutainer® sodium heparin tubes. Capillary blood was collected through a finger prick, while venous blood was drawn via standard venipuncture.

KHDSS

n=86

DBS Day 0

(Baseline)

n=85

DBS Day 30

(RT)

n=43

DBS Day 7

(RT)

n=42

Paired DBS (Filter and Mitra) and Plasma

n=263

MHDSS

n=90

NUHDSS

n=87

Plasma

(-80°C)

n=87

DBS Day 90

(RT)

n=87

DBS Day 90

(-20°C)

n=87

Plasma

(-80°C)

n=90

DBS >1 year

(RT)

n=90

DBS >1 year

(-20°C)

n=90

Plasma

(-80°C)

n=86

**Figure S2:** Collection of paired plasma and DBS samples using filter paper and Mitra devices. The DBS sample sizes collected from Kilifi (KHDSS), Nairobi Urban (NUHDSS) and Manyatta (MHDSS) sites are shown, along with storage conditions (room temperature (RT) or frozen) and corresponding elution time points for immunoassay analysis.

**Figure S3:** Relationship between age and log transformed plasma IgG concentrations across HDSS sites. Each point represents an individual participant, coloured according to HDSS site. smooth curves are fitted using locally weighted regression (LOESS). Solid lines represent locally weighted regression (LOESS) curves fitted separately for each site. Horizontal dashed lines indicate analyte-specific seropositivity thresholds.

**Figure S4:** Comparison of IgG antibody levels in DBS samples at baseline (day 0) with matched plasma. Correlation analysis of IgG concentrations in baseline DBS filter paper (red) and Mitra (blue) samples relative to plasma (n=85). The dotted diagonal line represents the reference lines (x=y), while the horizontal and vertical black dashed lines indicate the pathogen-specific seropositivity cut-off thresholds. Corresponding Pearson correlation coefficients (R) and concordance correlation coefficients (CCC) are shown.

**Figure S5:** Comparison of IgG antibody levels in DBS samples stored at -20°C relative to matched plasma samples. Correlation plots showing IgG concentrations quantified in DBS filter paper (red points and regression lines) and DBS Mitra (blue) compared with plasma (n=87) across seven analytes: diphtheria, tetanus, pertussis, measles, mumps, rubella, and varicella. The diagonal dotted line represents the x = y reference line. Horizontal and vertical black dashed lines indicate analyte-specific seropositivity cut-off thresholds. Panel **(A)** shows results from samples stored for 90 days, while panel **(B)** presents results from samples stored for >1 year. Pearson correlation coefficients (R) and concordance correlation coefficients (CCC) for each analyte and sample type are shown.

**Figure S6:** Analysis of IgG antibody levels in DBS samples stored at room temperature (RT) across various time points. Log-transformed antibody concentrations from DBS filter (upper panel) and Mitra (lower panel) eluates at RT day 7 (n=42) (**A**) and day 30 (n=43) (**B**) are compared with paired plasma levels. The line in each box represents the median, and the box shows the interquartile range (IQR). Whiskers indicate the range excluding outliers (>1.5× IQR). Black dotted lines mark pathogen-specific seropositivity cutoffs.

**Supplementary Tables**

Table S1: Age-adjusted differences in plasma IgG concentrations across HDSS sites relative to the Nairobi-Urban reference population. Fold changes were estimated using linear regression models fitted to log-transformed IgG concentrations.

| Analyte | HDSS site | Fold change (95% CI) | | p-value |
| --- | --- | --- | --- | --- |
| Diphtheria | Kilifi | 2.21 | (1.34−3.67) | <0.001 |
|  | Manyatta | 5.67 | (3.47−9.25) | <0.001 |
| Tetanus | Kilifi | 0.65 | (0.32−1.35) | 0.348 |
|  | Manyatta | 3.04 | (1.50−6.16) | <0.001 |
| Pertussis | Kilifi | 1.01 | (0.58−1.77) | 0.998 |
|  | Manyatta | 1.61 | (0.93−2.77) | 0.101 |
| Measles | Kilifi | 0.90 | (0.54−1.50) | 0.875 |
|  | Manyatta | 0.99 | (0.60−1.64) | 0.999 |
| Mumps | Kilifi | 0.75 | (0.51−1.12) | 0.208 |
|  | Manyatta | 1.16 | (0.79−1.71) | 0.62 |
| Rubella | Kilifi | 0.76 | (0.46−1.24) | 0.377 |
|  | Manyatta | 0.94 | (0.58−1.51) | 0.945 |
| Varicella | Kilifi | 0.43 | (0.25−0.73) | <0.001 |
|  | Manyatta | 1.09 | (0.65−1.85) | 0.916 |

Note: All sensitivity and specificity estimates are presented with 95% confidence intervals (95% CI).

**Table S2**: Sensitivity and Specificity of DBS filter and Mitra at baseline (Day 0) relative to plasma IgG responses (n = 85).

| Analyte | DBS Filter  Sensitivity | DBS Filter  Specificity | DBS Mitra  Sensitivity | DBS Mitra  Specificity |
| --- | --- | --- | --- | --- |
| Diphtheria | 100% (93.2−100) | 93.9% (79.8−99.3) | 96.2% (86.8−99.5) | 93.9% (79.8−99.3) |
| Tetanus | 95.8% (88.6−99.1) | 92.3% (64.0−99.8) | 98.6% (92.5−100) | 100% (75.3−100) |
| Pertussis | 72.2% (46.5−90.3) | 100% (94.6−100) | 77.8% (52.4−93.6) | 97.0% (89.6−99.6) |
| Measles | 96.0% (88.6−99.2) | 72.7% (39.0−94.0) | 100% (95.1−100) | 72.7% (39.0−94.0) |
| Mumps | 100% (95.3−100) | 89.0% (51.8−99.7) | 98.7% (92.9−100) | 100% (66.4−100) |
| Rubella | 100% (95.2−100) | 70.0% (34.8−93.3) | 100% (95.3−100) | 88.9% (51.8−99.7) |
| Varicella | 100% (93.7−100) | 89.3% (71.8−97.7) | 100% (93.7−100) | 96.4% (81.7−99.9) |

**Table S3**: Sensitivity and Specificity of DBS filter and Mitra at stored at -20°C for 90 days relative to plasma IgG responses (n = 87).

| Analyte | DBS Filter  Sensitivity | DBS Filter  Specificity | DBS Mitra  Sensitivity | DBS Mitra  Specificity |
| --- | --- | --- | --- | --- |
| Diphtheria | 90.9% (75.7−98.1) | 100% (93.4−100) | 93.9% (79.8−99.3) | 94.4% (84.6−98.8) |
| Tetanus | 100% (95.4−100) | 87.5% (47.4−99.7) | 100% (95.4−100) | 62.5% (24.5−91.5) |
| Pertussis | 62.5% (40.6−81.2) | 100% (94.6−100) | 87.5% (67.6−97.3) | 100% (94.3−100) |
| Measles | 93.8% (86.2−98.0) | 100% (54.1−100) | 96.3% (89.6−99.2) | 83.3% (35.9−99.6) |
| Mumps | 100% (95.7−100) | 75% (19.4−99.4) | 100% (95.7−100) | 100% (39.8−100) |
| Rubella | 98.8% (93.5−100) | 100% (29.2−100) | 100% (95.7−100) | 66.7% (9.4−99.2) |
| Varicella | 97.2% (90.3−99.7) | 97.2% (90.3−99.7) | 97.2% (90.3−99.7) | 93.3% (68.1−99.8) |

**Table S4**: Sensitivity and Specificity of DBS filter and Mitra at stored at -20°C for >1 year relative to plasma IgG responses (n = 90).

| Analyte | DBS Filter  Sensitivity | DBS Filter  Specificity | DBS Mitra  Sensitivity | DBS Mitra  Specificity |
| --- | --- | --- | --- | --- |
| Diphtheria | 77.0% (66.8−85.4) | 100% (2.5−100.0) | 95.4% (88.5−98.7) | 100% (2.5−100.0) |
| Tetanus | 97.6% (90.4−99.3) | N/A (0.0−100.0) | 100.0% (95.9−100.0) | N/A (0.0−100.0) |
| Pertussis | 33.3% (18.0−51.8) | 100% (93.5−100.0) | 71.9% (53.3−86.3) | 85.5% (73.3−93.5) |
| Measles | 81.6% (71.0−89.6) | 91.2% (61.5−99.8) | 92.1% (83.6−97.1) | 63.6% (30.1−89.1) |
| Mumps | 91.8% (83.8−96.6) | 100% (29.2−100.0) | 97.6% (91.7−99.7) | 0.0% (0.0−70.8) |
| Rubella | 96.4% (89.8−99.3) | 100.0% (47.8−100.0) | 100.0% (95.6−100.0) | 80.0% (28.4−99.5) |
| Varicella | 90.1% (80.7−95.9) | 94.1% (71.3−99.9) | 98.6% (92.3−100.0) | 88.2% (63.6−98.5) |

**Table S5:** Mixed‑effects model results for log₁₀‑transformed IgG concentrations across DBS storage durations. Models included storage duration as a categorical fixed effect and participant ID as a random intercept. Negative β estimates indicate lower IgG relative to the reference group; variance components are reported as participant‑level and residual SDs.

| Analyte | DBS matrix | Comparison | β Estimate | Std.  Error | 95% CI | P-value | Participant SD | Residual SD |
| --- | --- | --- | --- | --- | --- | --- | --- | --- |
| Diphtheria | Filter | Day 7 vs Day 0 | -0.035 | 0.022 | (-0.077 — 0.008) | 0.123 | 1.001 | 0.098 |
|  |  | Day 30 vs Day 0 | -0.100 | 0.066 | (-0.229 — 0.029) | 0.136 | 0.961 | 0.295 |
|  |  | Day 90 vs Plasma | -0.266 | 0.106 | (-0.474 — -0.058) | 0.014 | 0.705 | 0.700 |
|  |  | >1 year vs Plasma | -0.505 | 0.141 | (-0.782 — -0.229) | <0.001 | 0.259 | 0.935 |
|  | Mitra | Day 7 vs Day 0 | -0.077 | 0.048 | (-0.170 — 0.017) | 0.118 | 0.983 | 0.214 |
|  |  | Day 30 vs Day 0 | -0.180 | 0.064 | (-0.306 — -0.055) | 0.008 | 0.964 | 0.275 |
|  |  | Day 90 vs Plasma | -0.244 | 0.073 | (-0.388 — -0.101) | 0.001 | 0.870 | 0.483 |
|  |  | >1 year vs Plasma | -0.485 | 0.140 | (-0.759 — -0.212) | <0.001 | 0.300 | 0.925 |
|  | | | | | | | | |
| Tetanus | Filter | Day 7 vs Day 0 | -0.058 | 0.062 | (-0.180 — 0.065) | 0.362 | 0.967 | 0.279 |
|  |  | Day 30 vs Day 0 | -0.116 | 0.088 | (-0.288 — 0.056) | 0.195 | 0.925 | 0.393 |
|  |  | Day 90 vs Plasma | -0.341 | 0.092 | (-0.521 — -0.161) | <0.001 | 0.781 | 0.606 |
|  |  | >1 year vs Plasma | -0.807 | 0.126 | (-1.054 — -0.560) | <0.001 | 0.375 | 0.837 |
|  | Mitra | Day 7 vs Day 0 | -0.101 | 0.060 | (-0.219 — 0.016) | 0.099 | 0.969 | 0.269 |
|  |  | Day 30 vs Day 0 | -0.223 | 0.070 | (-0.360 — -0.086) | 0.003 | 0.954 | 0.301 |
|  |  | Day 90 vs Plasma | -0.293 | 0.060 | (-0.411 — -0.175) | <0.001 | 0.909 | 0.398 |
|  |  | >1 year vs Plasma | -0.762 | 0.123 | (-1.003 — -0.521) | <0.001 | 0.437 | 0.816 |
|  | | | | | | | | |
| Pertussis | Filter | Day 7 vs Day 0 | -0.058 | 0.028 | (-0.112 — -0.003) | 0.044 | 0.998 | 0.124 |
|  |  | Day 30 vs Day 0 | -0.195 | 0.062 | (-0.316 — -0.074) | 0.003 | 0.963 | 0.276 |
|  |  | Day 90 vs Plasma | -0.243 | 0.079 | (-0.398 — -0.089) | 0.003 | 0.849 | 0.520 |
|  |  | >1 year vs Plasma | -0.691 | 0.136 | (-0.957 — -0.424) | <0.001 | 0.258 | 0.905 |
|  | Mitra | Day 7 vs Day 0 | -0.180 | 0.053 | (-0.283 — -0.076) | 0.002 | 0.974 | 0.237 |
|  |  | Day 30 vs Day 0 | -0.219 | 0.063 | (-0.342 — -0.096) | 0.001 | 0.964 | 0.269 |
|  |  | Day 90 vs Plasma | -0.238 | 0.074 | (-0.383 — -0.093) | 0.002 | 0.868 | 0.488 |
|  |  | >1 year vs Plasma | -0.608 | 0.125 | (-0.852 — -0.364) | <0.001 | 0.478 | 0.827 |
|  | | | | | | | | |
| Measles | Filter | Day 7 vs Day 0 | -0.016 | 0.014 | (-0.044 — 0.012) | 0.263 | 1.004 | 0.064 |
|  |  | Day 30 vs Day 0 | -0.187 | 0.057 | (-0.300 — -0.075) | 0.002 | 0.969 | 0.256 |
|  |  | Day 90 vs Plasma | -0.450 | 0.082 | (-0.611 — -0.289) | <0.001 | 0.814 | 0.541 |
|  |  | >1 year vs Plasma | -0.422 | 0.136 | (-0.689 — -0.155) | 0.003 | 0.374 | 0.906 |
|  | Mitra | Day 7 vs Day 0 | -0.118 | 0.055 | (-0.225 — -0.011) | 0.037 | 0.974 | 0.245 |
|  |  | Day 30 vs Day 0 | -0.257 | 0.111 | (-0.474 — -0.040) | 0.026 | 0.878 | 0.476 |
|  |  | Day 90 vs Plasma | -0.348 | 0.074 | (-0.493 — -0.202) | <0.001 | 0.857 | 0.490 |
|  |  | >1 year vs Plasma | -0.398 | 0.137 | (-0.667 — -0.128) | 0.005 | 0.366 | 0.912 |
|  | | | | | | | | |
| Mumps | Filter | Day 7 vs Day 0 | -0.027 | 0.074 | (-0.172 — -0.118) | 0.716 | 0.950 | 0.331 |
|  |  | Day 30 vs Day 0 | -0.192 | 0.096 | (-0.379 — -0.005) | 0.051 | 0.906 | 0.427 |
|  |  | Day 90 vs Plasma | -0.203 | 0.100 | (-0.398 — -0.008) | 0.045 | 0.751 | 0.657 |
|  |  | >1 year vs Plasma | -1.009 | 0.126 | (-1.256 — -0.763) | <0.001 | 0.216 | 0.837 |
|  | Mitra | Day 7 vs Day 0 | -0.115 | 0.055 | (-0.222 — -0.007) | 0.043 | 0.974 | 0.245 |
|  |  | Day 30 vs Day 0 | -0.172 | 0.072 | (-0.313 — -0.032) | 0.021 | 0.955 | 0.308 |
|  |  | Day 90 vs Plasma | -0.255 | 0.064 | (-0.381 — -0.129) | <0.001 | 0.899 | 0.425 |
|  |  | >1 year vs Plasma | -0.923 | 0.128 | (-1.175 — -0.672) | <0.001 | 0.270 | 0.848 |
|  | | | | | | | | |
| Rubella | Filter | Day 7 vs Day 0 | -0.020 | 0.036 | (-0.089 — 0.050) | 0.585 | 0.994 | 0.159 |
|  |  | Day 30 vs Day 0 | -0.163 | 0.063 | (-0.286 — -0.040) | 0.013 | 0.963 | 0.281 |
|  |  | Day 90 vs Plasma | -0.690 | 0.089 | (-0.864 — -0.517) | <0.001 | 0.738 | 0.584 |
|  |  | >1 year vs Plasma | -1.123 | 0.120 | (-1.358 — -0.887) | <0.001 | 0.221 | 0.799 |
|  | Mitra | Day 7 vs Day 0 | -0.153 | 0.066 | (-0.282 — -0.023) | 0.026 | 0.959 | 0.295 |
|  |  | Day 30 vs Day 0 | -0.348 | 0.087 | (-0.519 — -0.177) | <0.001 | 0.918 | 0.375 |
|  |  | Day 90 vs Plasma | -0.539 | 0.075 | (-0.686 — -0.391) | <0.001 | 0.829 | 0.495 |
|  |  | >1 year vs Plasma | -1.044 | 0.110 | (-1.260 — -0.828) | <0.001 | 0.439 | 0.731 |
|  | | | | | | | | |
| Varicella | Filter | Day 7 vs Day 0 | -0.087 | 0.047 | (-0.179 — 0.005) | 0.071 | 0.983 | 0.210 |
|  |  | Day 30 vs Day 0 | -0.147 | 0.045 | (-0.235 — -0.058) | 0.002 | 0.983 | 0.202 |
|  |  | Day 90 vs Plasma | -0.470 | 0.087 | (-0.640 — -0.301) | <0.001 | 0.789 | 0.572 |
|  |  | >1 year vs Plasma | -0.905 | 0.102 | (-1.104 — -0.705) | <0.001 | 0.582 | 0.675 |
|  | Mitra | Day 7 vs Day 0 | -0.109 | 0.036 | (-0.179 — -0.040) | 0.004 | 0.992 | 0.159 |
|  |  | Day 30 vs Day 0 | -0.235 | 0.066 | (-0.365 — -0.104) | 0.001 | 0.958 | 0.286 |
|  |  | Day 90 vs Plasma | -0.450 | 0.072 | (-0.591 — -0.308) | <0.001 | 0.853 | 0.477 |
|  |  | >1 year vs Plasma | -0.844 | 0.088 | (-1.017 — -0.672) | <0.001 | 0.695 | 0.583 |

**Table S6**: Sensitivity and Specificity of DBS filter and Mitra at Day 7 room temperature storage relative to Day 0 time point IgG responses (n = 42).

| Analyte | DBS Filter  Sensitivity | DBS Filter  Specificity | DBS Mitra  Sensitivity | DBS Mitra  Specificity |
| --- | --- | --- | --- | --- |
| Diphtheria | 95.2% (76.2−99.9) | 100% (82.4−100) | 95.5% (77.2−99.9) | 100% (81.5−100) |
| Tetanus | 97.1% (84.7−99.9) | 83.3% (35.9−99.6) | 100% (89.7−100) | 100% (54.1−100) |
| Pertussis | 71.4% (29.0−96.3) | 100% (89.4−100) | 66.7% (29.9−92.5) | 100% (88.8−100) |
| Measles | 100% (90.0−100) | 80.0% (28.4−99.5) | 97.3% (85.8−99.9) | 100% (29.2−100) |
| Mumps | 97.2% (85.5−99.9) | 100% (39.8−100) | 100% (90.3−100) | 100% (39.8−100) |
| Rubella | 97.2% (85.5−99.9) | 100% (39.8−100) | 97.2% (85.5−99.9) | 100% (39.8−100) |
| Varicella | 100% (89.1−100) | 100% (63.1−100) | 96.9% (83.8−99.9) | 100% (63.1−100) |

**Table S7**: Sensitivity and Specificity of DBS filter and Mitra at Day 30 room temperature storage relative to Day 0 time point IgG responses (n = 43).

| Analyte | DBS Filter  Sensitivity | DBS Filter  Specificity | DBS Mitra  Sensitivity | DBS Mitra  Specificity |
| --- | --- | --- | --- | --- |
| Diphtheria | 96.4% (81.7−99.9) | 100% (73.6−100) | 92.0% (74.0−99.0) | 100% (73.5−100) |
| Tetanus | 91.2% (76.3−98.1) | 100% (54.1−100) | 100% (88.4−100) | 71.4% (29.0−96.3) |
| Pertussis | 75.0% (34.9−96.8) | 96.9% (83.8−99.9) | 60.0% (14.7−94.7) | 100% (89.1−100) |
| Measles | 96.9% (83.8−99.9) | 62.5% (24.5−91.5) | 97.3% (85.8−99.9) | 100% (29.2−100) |
| Mumps | 100% (90.3−100) | 100% (39.8−100) | 93.3% (77.9−99.2) | 85.7% (42.1−99.6) |
| Rubella | 100% (89.7−100) | 100% (54.1−100) | 100% (89.1−100) | 100% (47.8−100) |
| Varicella | 100% (85.8−100) | 93.8% (69.8−99.8) | 100% (83.2−100) | 100% (80.5−100) |

**Table S8:** Sensitivity and Specificity of DBS filter and Mitra at stored at Room Temperature (RT) for 90 days relative to plasma IgG responses (n = 87).

| Analyte | DBS Filter  Sensitivity | DBS Filter  Specificity | DBS Mitra  Sensitivity | DBS Mitra  Specificity |
| --- | --- | --- | --- | --- |
| Diphtheria | 69.7% (51.3−84.4) | 98.2% (90.1−100) | 70.3% (53.02−84.1) | 100% (92.9−100) |
| Tetanus | 96.2% (89.3−99.2) | 75.0% (34.9−96.8) | 98.7% (93.2−100) | 62.5% (24.5−91.5) |
| Pertussis | 62.5% (40.6 − 81.2) | 100% (94.6−100) | 42.9% (21.8−66.0) | 100% (94.6−100) |
| Measles | 45.8% (25.6−67.2) | 100% (94.3−100) | 84.2% (74.4−91.3) | 100% (47.8−100) |
| Mumps | 100% (95.7−100) | 75.0% (19.4−99.4) | 96.4% (89.8−99.3) | 50.0% (6.8−93.2) |
| Rubella | 76.5% (65.8−85.3) | 100% (54.1−100) | 94.2% (87.0−98.1) | 100% (2.5−100) |
| Varicella | 97.2% (90.3−99.7) | 97.2% (90.3−99.7) | 88.9% (79.3−95.1) | 93.3% (68.1−99.8) |

**Table S9:** Sensitivity and Specificity of DBS filter and Mitra stored at RT for >1 year relative to plasma IgG responses (n = 90).

| Analyte | DBS Filter  Sensitivity | DBS Filter  Specificity | DBS Mitra  Sensitivity | DBS Mitra  Specificity |
| --- | --- | --- | --- | --- |
| Diphtheria | 5.9% (1.9−13.2) | 100% (2.5−100.0) | 10.6% (5.0−19.1) | 100.0% (2.5−100.0) |
| Tetanus | 57.5% (46.4−68.0) | _% (0.0−100.0) | 69.0% (58.1−78.5) | _% (0.0−100.0) |
| Pertussis | 3.1% (0.1−16.2) | 100.0% (93.5−100.0) | 25.0% (11.5−43.4) | 100.0% (93.5−100.0) |
| Measles | 21.3% (12.7−32.3) | 100.0% (73.5−100.0) | 29.0% (19.1−40.5) | 100.0% (71.5−100.0) |
| Mumps | 19.1% (11.3−29.1) | 100.0% (29.2−100.0) | 34.2% (24.0−45.5) | 66.7% (9.4−99.2) |
| Rubella | 29.3% (19.7−40.4) | 100.0% (47.8−100.0) | 46.3% (35.3−57.7) | 100.0% (47.8−100.0) |
| Varicella | 10.0% (4.1−19.5) | 100.0% (80.5−100.0) | 24.3% (14.8−36.0) | 100.0% (80.5−100.0) |
